# Culturable Diversity and Lipid Production Profile of Labyrinthulomycete Protists Isolated from Coastal Mangrove Habitats of China

**DOI:** 10.3390/md17050268

**Published:** 2019-05-06

**Authors:** Qiuzhen Wang, Huike Ye, Yunxuan Xie, Yaodong He, Biswarup Sen, Guangyi Wang

**Affiliations:** 1 Center for Marine Environmental Ecology, School of Environmental Science and Engineering, Tianjin University, Tianjin 300072, China; qqzz1990@tju.edu.cn (Q.W.); yehuike@163.com (H.Y.); yxie@tju.edu.cn (Y.X.); yaodong.he@tju.edu.cn (Y.H.); bsen@tju.edu.cn (B.S.); 2 Ocean College of Hebei Agricultural University, Qinhuangdao 066000, China; 3 Key Laboratory of Systems Bioengineering (Ministry of Education), Tianjin University, Tianjin 300072, China

**Keywords:** lipid profiling, thraustochytrids, labyrinthulids, isolation, phylogeny, batch culture, optimization

## Abstract

Labyrinthulomycete protists have gained significant attention in the recent past for their biotechnological importance. Yet, their lipid profiles are poorly described because only a few large-scale isolation attempts have been made so far. Here, we isolated more than 200 strains from mangrove habitats of China and characterized the molecular phylogeny and lipid accumulation potential of 71 strains. These strains were the closest relatives of six genera namely *Aurantiochytrium*, *Botryochytrium*, *Parietichytrium*, *Schizochytrium*, *Thraustochytrium*, and *Labyrinthula*. Docosahexaenoic acid (DHA) production of the top 15 strains ranged from 0.23 g/L to 1.14 g/L. Two labyrinthulid strains, GXBH-107 and GXBH-215, exhibited unprecedented high DHA production potential with content >10% of biomass. Among all strains, ZJWZ-7, identified as an *Aurantiochytrium* strain, exhibited the highest DHA production. Further optimization of culture conditions for strain ZJWZ-7 showed improved lipid production (1.66 g/L DHA and 1.68 g/L saturated fatty acids (SFAs)) with glycerol-malic-acid, peptone-yeast-extract, initial pH 7, 28 °C, and rotation rate 150 rpm. Besides, nitrogen source, initial pH, temperature, and rotation rate had significant effects on the cell biomass, DHA, and SFAs production. This study provides the identification and characterization of nearly six dozen thraustochytrids and labyrinthulids with high potential for lipid accumulation.

## 1. Introduction

Labyrinthulomycete protists are a group of marine fungus-like organisms classified within the kingdom Straminipila and are characterized by their ability to produce heterokont biflagellate zoospores and ectoplasmic networks [1]. Based on their morphological and molecular characteristics, these osmoheterotrophic protists are grouped into three distinct groups namely thraustochytrids, labyrinthulids, and aplanochytrids [2,3,4,5]. These protists live as commensals or mutualists within the guts and tissues of marine invertebrates, and also as saprobes on mollusk shells and their feces [5]. They are considered to play important roles toward the decomposition and mineralization of organic detritus, such as macroalgae and submerged mangrove leaves, by producing a range of hydrolytic enzymes [6,7,8]. By production of motile zoospores, these heterotrophic protists colonize detritus much more rapidly than fungi and play significant roles in the aquatic food web through degradative activities [8]. Partial evidence for the ecological roles of this group of protists suggests their significance in the coastal food-web dynamics and organic matter cycling [9]. 

Labyrinthulomycetes are the most ubiquitous and widespread straminipilan protists in oceans with a total of 13 known genera and 30 described species so far [10]. They have been isolated, using the direct-plating and pollen-baiting methods, from various habitats (e.g., marine seawater, estuarine water, sediments, decaying mangrove leaves, and senescent macroalgae) [11,12,13,14,15,16]. Yet, the majority of the species described are isolated from only a limited number of coastal regions, for example, Japan [17], Australia [12,18], Canada [19], India [20,21,22], Chile [11], China [23], Malaysia [24,25], and Thailand [26]. Consequently, the catalogue of these protists for many of the coastal regions across the globe remain poorly described, which limits our understanding of their true culturable diversity and ecological significance. 

Although the labyrinthulids group are important ecologically, there is no formal estimate of their culturable diversity. So far, only a few studies have reported on the isolation of labyrinthulids [15,27]. On the contrary, the group thraustochytrids has gained significant attention of researchers because of their biotechnological potential [28]. Especially, some species of *Schizochytrium* and *Aurantiochytrium* exhibit the ability to produce large amounts of polyunsaturated fatty acids (PUFAs) such as docosahexaenoic acid (DHA) used in dietary supplements [13,24,25,29,30,31,32,33]. Besides DHA, some strains of thraustochytrids (e.g., *Schizochytrium* sp. PKU#Mn4 and *Thraustochytriidae* sp. PKU#Mn16 were reported to produce high amounts of saturated fatty acids (SFAs) [34]. While the thraustochytrids group comprises well-known DHA and SFAs producers, only one report suggests the potential of labyrinthulid protists for DHA production [27]. Despite growing evidence for potential PUFAs and SFAs production, the culturable diversity and optimal culture conditions of thraustochytrids and labyrinthulids are yet to be fully understood. 

**Figure 1:**
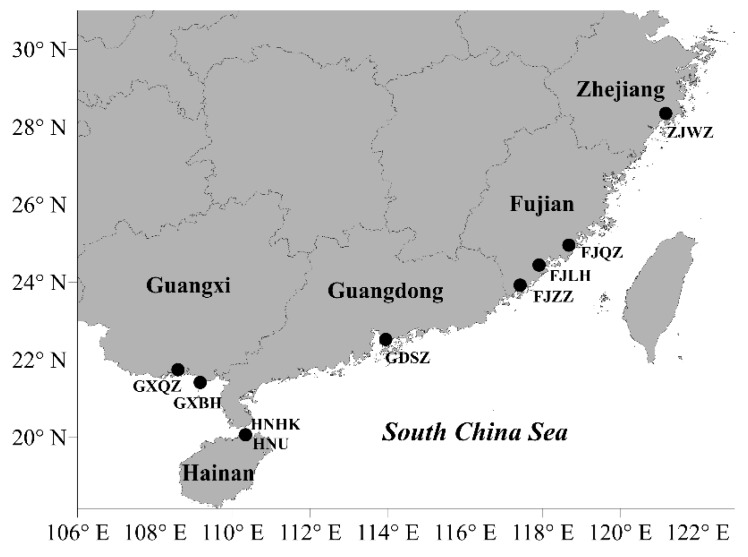
Distribution of sampling sites in the mangrove habitats of China.

China has a long coastline spanning the Yellow Sea, East China Sea, and the South China Sea. However, up until now, only 60 thraustochytrid strains, closely related to the genera *Schizochytrium*, *Aurantiochytrium*, and *Thraustochytrium*, have been isolated from the coastal waters of China (i.e., Shenzhen bay) [23]. Therefore, in this study, we conducted isolation of Labyrinthulomycete protists on large-scale covering sampling sites from five different coastal provinces of China (Figure 1). Besides phylogenetic diversity across these provinces, this study reports the DHA production potential of representative thraustochytrid and labyrinthulid isolates and the optimal culture conditions for maximal DHA accumulation by the top strain. The present investigation aims to provide a comprehensive report on the isolation, characterization, phylogeny, and lipid production potential of several dozen Labyrinthulomycetes from Chinese coastal habitats.

## 2. Results

### 2.1. Culturable Diversity of Labyrinthulomycete Protists

More than 200 Labyrinthulomycete isolates were obtained from marine samples (mangrove leaves covered with sediment and water) collected from the coastal habitats of China (Figure 1, Table 1) using both direct plating and pine-pollen baiting methods. Colonies of all the isolates displayed whitish appearance. Under the microscope, the thraustochytrid cells exhibited globose or sub-globose shape while labyrinthulid cells appeared spindle-shaped (Figure S1). From the primary screening based on the Nile Red staining method, a total of 71 strains were found to accumulate lipid. Partial 18S rRNA gene sequence analysis placed these strains into two major groups: thraustochytrids and labyrinthulids (Table 1 and Table S1). At the genus level, the closest relatives of the 71 strains belonged to six different genera namely *Aurantiochytrium*, *Botryochytrium*, *Parietichytrium*, *Schizochytrium*, *Thraustochytrium*, and *Labyrinthula* (Table 2). Of these six genera, *Botryochytrium*, *Pareietichytrium*, and *Labyrinthula* are reported for the first time in the coastal waters of China. *Aurantiochytrium, Schizochytrium*, and *Labyrinthula* were the three most dominant genera that accounted for ca. 61%, 21.7%, and 13.2% of the total isolates, respectively (Table 2). Low abundance genera, *Parietichytrium*, *Botryochytrium*, and *Thraustochytrium*, were represented by only two, one, and six strains. All the six genera isolated in this research were detected in Hainan province. Four genera namely *Aurantiochytrium*, *Schizochytrium*, *Thraustochytrium,* and *Labyrinthula* were isolated in Guangxi province, while *Parietichytrium* and *Thraustochytrium* were the only two genera isolated in Guangdong province. Notably, only *Aurantiochytrium* strains were isolated in Fujian and Zhejiang provinces. Overall, about 93% of the total strains were isolated from Guangxi and Hainan provinces and only three strains were isolated from Fujian and Zhejiang provinces.

**Figure 2:**
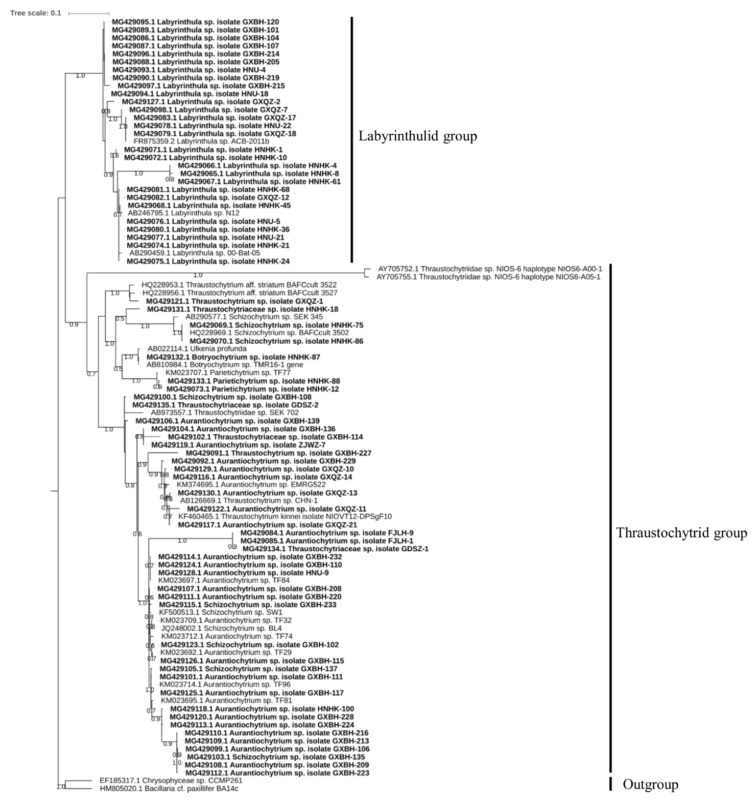
A maximum-likelihood phylogenetic tree of Labyrinthulomycetes isolated from coastal mangrove areas of China. The branch lengths of the tree are represented by the “Tree scale” shown in the upper-left corner.

Phylogenetic analysis of the 71 strains revealed that the thraustochytrid and labyrinthulid clades are monophyletic and had diversified from a common ancestor (Figure 2). The strains belonging to genera *Aurantiochytrium*, *Botryochytrium*, *Parietichytrium*, *Schizochytrium*, *Thraustochytrium* separated into four groups with a paraphyletic relationship to each other. These four paraphyletic groups included: group 1 (*Thraustochytrium* sp. GXQ2-1), group 2 (*Thraustochytriaceae* sp. HNHK-18, *Schizochytrium* sp. HNHK-75 and HNHK-86), group 3 (*Botryochytrium* sp. HNHK-87, *Parietichytrium* sp. HNHK-88 and HNHK-12), and group 4 (*Thraustochytriaceae* sp. GDSZ-2, *Schizochytrium* sp. GXBH-108, and all strains of *Aurantiochytrium*). The two strains each of *Thraustochytriaceae* sp. (HNHK-18 and GDSZ-2) and *Schizochytrium* sp. (HNHK-75 and GXBH-108) were distributed in different groups, which suggest that the currently used nomenclature might need reconsideration. Overall, our ML tree of thraustochytrid and labyrinthulid groups clearly demonstrate their monophyletic relationship consistent with the previous report [35].

### 2.2. Screening for High Docosahexaenoic Acid (DHA)-Producing Strains

**Figure 3:**
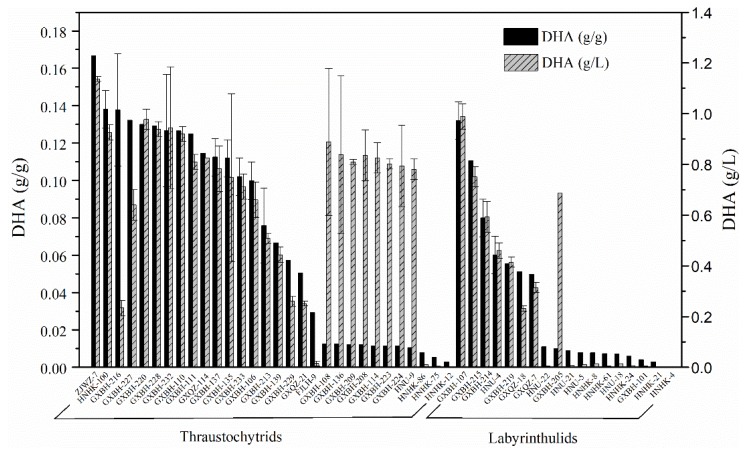
The DHA production profile of potential strains of thraustochytrids and labyrinthulids.

The lipid accumulation in representatives of 71 thraustochytrid and labyrinthulid strains was semi-quantitatively analyzed by Nile Red staining method (Figure S1). Subsequently, a total of 48 strains with the capacity for intracellular lipid accumulation were identified. Of these 48 strains, 30 were members of the thraustochytrid group while 18 strains belonged to the labyrinthulid group (Figure 3). The results of growth and DHA production of these screened strains demonstrated that DHA content of 15 strains, including 13 thraustochytrid and two labyrinthulid strains, reached up to 10% of biomass (Table 3). The DHA yields of these 15 potential DHA-producing strains differed widely, ranging from 0.23 g/L to 1.14 g/L (Figure 3). Among these strains, the maximum PUFAs (% total fatty acids (TFAs)) and DHA content (% TFAs) were 53.69% and 42.95%, respectively, in strain GXBH-216. However, this strain had limited biotechnological applications because of its low biomass yield (1.72 g/L) (Table 3). On the other hand, GXBH-220 although showed the highest biomass yield (7.52 g/L), had a lower TFAs content (30.69% biomass). Similarly, strain GXBH-227 which exhibited the highest TFAs content (47.57% biomass) showed the lowest DHA content (28.54% TFAs). Notably, strain ZJWZ-7 isolated from subtropical coastal mangroves of Wenzhou in Zhejiang province showed the highest DHA (0.17 g/g and 1.14 g/L) and PUFAs (20.35% biomass and 1.39 g/L) production. Therefore, further optimization of the culture conditions for strain ZJWZ-7 was conducted with the objective of improving its DHA accumulation.

The fatty acids profiles of the 15 strains revealed that SFAs, monounsaturated fatty acids (MUFAs), and PUFAs were the major constituents (Table 3). The dominant fatty acids in these strains were palmitic acid (PA, C16:0, 36.11%–46.32%) and DHA (C22:6n3, 28.54%–42.95%). The other fatty acids present in relatively fewer amounts were docosapentaenoic acid (DPA, C22:5n3, ~7.42%–9.77%), myristic acid (MA, C14:0, ~3.90%–5.01%), and pentadecanoic acid (C15:0, ~1.54%–4.58%). Besides dodecanoic acid (C12:0), heptadecanoic acid (C17:0), octadecanoic acid (C18:0), octadecenoic acid (C18:1n9), arachidonic acid (ARA, C20:4n6), and eicosapentaenoic acid (EPA, C20:5n3) were also detected at low levels in this study.

### 2.3. Effect of Culture Conditions on the Cell Biomass, DHA and Saturated Fatty Acids (SFAs) Production

**Figure 4:**
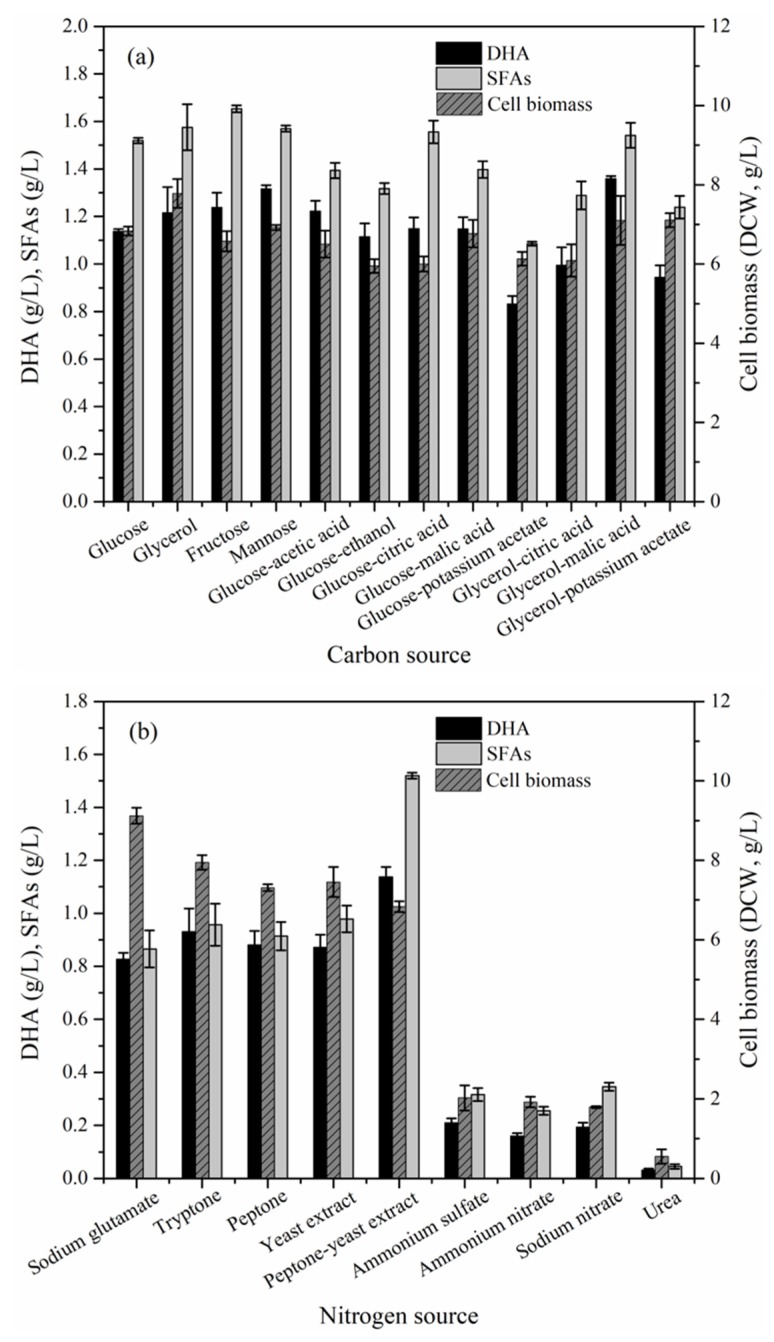
The cell biomass, DHA and SFAs production of *Aurantiochytrium* sp. ZJWZ-7 under varying (**a**) carbon sources and (**b**) nitrogen sources. Data are shown for samples collected at 72 h of cultivation.

The cell biomass, DHA and SFAs production of strain ZJWZ-7 increased with incubation time under the initial flask culture conditions. Their peak values were observed after 72 h (stationary stage) of cultivation (Figure S2). The one-factor-at-a-time (OFAT) optimization of the initial culture conditions for strain ZJWZ-7 demonstrated significant effects (*P* < 0.05) of nitrogen source, temperature, initial pH, and rotation rate on the cell biomass, DHA and SFAs production (Table 4). Although carbon source had an insignificant effect (*P* > 0.05) on the SFAs production, it showed a significant (*P* < 0.05) effect on the biomass and DHA production (Table 4). Of all the carbon sources tested, glycerol-malic-acid and fructose were the best for DHA and SFAs production, respectively (Figure 4a). On the other hand, peptone-yeast-extract was the best nitrogen source for DHA and SFAs production (Figure 4b). In general, the organic nitrogen, except urea, facilitated the cell growth and DHA accumulation more effectively than the inorganic nitrogen (Figure 4b). In addition, the OFAT experimental results revealed that the optimization of KH_2_PO_4_ and salinity may not significantly (P>0.05) improve the cell growth, DHA and SFAs production of strain ZJWZ-7 (Table 5). Notably, DHA production by strain ZJWZ-7 was relatively more at low temperature (25–28 °C), initial pH (pH 7), and rotating rate (150–250 rpm) (Table 5). 

Overall, the optimal culture conditions, (i.e., glycerol-malic-acid as a carbon source, peptone-yeast-extract as a nitrogen source, initial pH 7, 28 °C, and rotation rate 150 rpm) improved DHA production by strain ZJWZ-7.

### 2.4. Batch Production of DHA by Strain ZJWZ-7 under Optimal Conditions

In order to validate the optimal culture conditions derived from OFAT optimization, the batch culture of strain ZJWZ-7 conducted in 500 mL flask was studied (Table 6). With respect to the initial culture conditions, the cell biomass, PUFAs and DHA production increased significantly by 44.66% (from 6.83 g/L to 9.88 g/L), 43.88% (from 1.39 to 2.00 g/L) and 45.61% (from 1.14 g/L to 1.66 g/L), respectively, in optimal batch culture. Although SFAs production did not increase much with reference to the initial conditions, the PUFAs content (% TFAs) increased from 46.07% to 52.97%. DHA was the dominant PUFA which increased up to 44.52% (% TFAs) in optimal batch culture. C16:0 accounted for 37.63% followed by DPA (7.57%), MA (3.28%), and pentadecanoic acid (1.89%). Other minor fatty acids (≤1% TFAs) included dodecanoic acid, octadecanoic acid, ARA, and EPA.

## 3. Discussion

### 3.1. Isolation and Phylogenetic Diversity of Labyrinthulomycetes

In this study, two standard cultivation methods, the direct plating and baiting, combined with some modifications were employed for the isolation of thraustochytrids and labyrinthulids from different coastal mangrove habitats in China. Although these methods have been widely used as two basic techniques for the isolation of thraustochytrids and labyrinthulids, some cells observed under microscopy in both the fresh samples and the colonized baits could not be isolated successfully by these methods. Therefore, standard procedures were modified according to the nature of the isolates in our study. Consequently, more than 200 thraustochytrid and labyrinthulid strains were obtained using MC medium complexed with ampicillin, streptomycin, and nystatin. Several lines of evidence suggest the use of diverse baits (pine pollen, sweet gum, and shrimp larvae), nutrient composition, agar concentration of the medium, antibiotics combination (ampicillin, streptomycin, chloramphenicol, kanamycin, and tetracycline) and concentration as key factors for successful isolation [36,37,38]. Besides these factors, mimicking the natural environment, covering the colonies with sterilized seawater for sporulation [39], use of incubation temperature similar to that in natural habitats, repeat inoculation and successive observation under a microscope are also considered critical for isolation of Labyrinthulomycete protists [38]. 

Labyrinthulomycetes have been isolated and identified from tropical, sub-tropical, temperate and cold areas, including India, Philippines, Hong Kong, Japan, China, New Zealand, Chile, Australia, Brazil, and so on [15,23,38,40,41]. In China, several studies have been conducted to investigate the culturable diversity of thraustochytrids, mainly from coastal waters of Shenzhen and Hong Kong mangroves [23,40]. Until now, only five genera of Thraustochytrids, i.e., *Aurantiochytrium*, *Schizochytrium*, *Thraustochytrium*, *Aplanochytrium*, and *Oblongichytrium* have been reported to be isolated from mangroves or seawaters in China [23]. In this study, we provide the first report of the isolation of several labyrinthulid strains from Chinese coastal habitats. 

### 3.2. Screening for High DHA Production Strains

Thraustochytrids are well-known marine heterotrophic protists for their high DHA-production potential [28]. Most of the thraustochytrid strains studied for the DHA production in previous research were isolated from tropical and sub-tropical regions [11,12,14,15,16,18,20,21,23,25,40,41,42]. Only a few studies focused on DHA production in thraustochytrids from temperate habitats [13,17,43,44]. It has been suggested that microorganisms isolated from a low-temperature environment tend to produce more PUFAs [45]. Recently, thraustochytrid strains with high relative levels of omega-3 PUFAs were isolated from the North Sea region of British waters including one particular strain TL18 with high DHA content (67% of TFA) [44]. Similar to the earlier report from cold habitat, our strain ZJWZ-7 isolated from the temperate habitat of China exhibited the highest DHA production (0.17 g/g, 1.14 g/L). On the other hand, numerous studies have demonstrated that *Aurantiochytrium* and *Schizochytrium* sp. are particularly good candidates for DHA production [46,47,48,49]. In this study, 48 strains with high lipid accumulation were screened by Nile Red staining method, and 15 high DHA producing (>10% biomass) strains were characterized. More than half of the 15 high-DHA-producing strains belonged to *Aurantiochytrium,* which suggest a high DHA-producing characteristic of this genus. 

With regard to labyrinthulids, most research was concentrated on their pathogenic effect on the seagrass [50,51], and only a few were about their DHA production [15,27]. Up to now only one labyrinthulid strain, L72, isolated from a fallen leaf in the Seto Inland Sea of Japan is reported that produces only DHA among all the long-chain PUFAs [27]. In the present study, two newly isolated labyrinthulid strains, GXBH-107 and GXBH- 215, exhibited high DHA production (>10% biomass). This is the first report on the determination of DHA production in labyrinthulid strains isolated from Chinese coastal habitats. Future studies on their ecophysiology and optimal culture conditions should provide evidence of their biotechnological potential.

### 3.3. Optimization and Validation of the Culture Conditions

Cell growth and lipid accumulation in thraustochytrids during fermentation can be affected by several factors [52,53,54]. The optimization of media nutrients and environmental conditions not only improves cell growth and DHA production but also allows the selection of alternative feedstock. For example, several renewable and waste materials as substrates for DHA production by thraustochytrids are reported, including biodiesel-derived crude glycerol, stalk juice of sweet sorghum, and residues from beer and potato [55,56,57]. In the present study, glycerol proved to be the best carbon source for cell growth and DHA production, which demonstrates the potential of the strain ZJWZ-7 towards DHA production using biodiesel-derived glycerol. Compared to inorganic nitrogen sources, strain ZJWZ-7 showed a general preference for organic nitrogen sources. Furthermore, a combination of peptone and yeast extract showed the highest potential for DHA production (0.11 g/g, 0.77 g/L), probably because of the presence of amino acids and vitamins which are known to promote cell growth and DHA accumulation. These findings are consistent with previous studies which reported the better action of organic than inorganic nitrogen sources on lipid production [33,34,54]. Our results provide evidence that a combined organic nitrogen source can significantly improve DHA production over a single nitrogen source. In addition to carbon and nitrogen sources, temperature, initial pH and rotation rate are also known to exhibit a significant effect on DHA production, and our results are consistent with the earlier findings [33,54]. However, consistent with a previous report [33], we did not observe a significant effect of KH_2_PO_4_ and salinity on DHA production by the strain ZJWZ-7 in the present study. This suggests that at low salinity and rotation rate, high cell biomass and DHA production could be achieved, which can be beneficial for an economical fermentation process on a larger scale. 

In the present study, the best medium components and environmental conditions were obtained by OFAT optimization in flask culture and subsequently applied on a larger scale. DHA turned out to be the predominant fatty acid and its representation in total fatty acids increased to 44.52% from 37.68% (as noted in flask culture). Other PUFAs, such as ARA, EPA, and DPA were also produced by strain ZJWZ-7, which show various beneficial effects on human health and can only be absorbed from the daily diet by the human body. On the other hand, high level of SFAs production (44.4% in TFAs and 1.68 g/L) was achieved simultaneously under the optimal conditions for DHA production, which can be valuable by-products for biodiesel production. Overall, our study provides evidence which indicates that ZJWZ-7 could be a potential strain for both DHA and SFAs production in industrial-scale fermentation.

## 4. Materials and Methods

### 4.1. Sample Collection and Strain Isolation

Mangrove leaves covered by sediments and seawater were collected from Zhejiang, Fujian, Guangdong, Guangxi, and Hainan provinces of China between July 2015 and April 2016 for the isolation of Labyrinthulomycetes (Figure 1; Table 1). Samples were collected into sterile plastic tubes and were immediately brought back to the laboratory for isolation, as described in our previous study [23]. Both direct plating and pine-pollen baiting were used for the isolation in this study. Sediments mixed with seawater on the mangrove leaves were directly streaked and spread on the modified Mar Chiquita (MC) medium containing 2 g/L glucose, 1 g/L peptone, 1 g/L yeast extract, 1 g/L sodium glutamate, 1 g/L corn steep liquor, 20 g/L agar and 33 g/L artificial sea salt [38]. Mangrove leaves were cut into small fragments of 0.5–1 cm diameter, some of which were washed with sterile artificial seawater, and were then placed directly on the MC medium. The other pieces of leaf fractions were immersed in sterile artificial seawater with a small amount of sterilized pine pollen scattered on its surface to attract Labyrinthulomycetes. These samples were then incubated at room temperature and observed daily with a microscope for 2–4 days to enrich the isolates. The pine pollen covered with the microorganisms was subsequently picked and sub-cultured on MC medium containing antibiotics (0.05% ampicillin, 0.075% streptomycin, and 0.005% nystatin) [23]. These MC media were incubated at room temperature with regular observation until colonies appeared. The resulting colonies were transferred to the fresh MC medium containing antibiotics (0.05% ampicillin, 0.075% streptomycin, and 0.005% nystatin) for purification. After several times of streak cultivation, the pure isolates were obtained which were then inoculated in 1% modified Vishniac’s (MV) agar plates containing 10 g/L glucose, 1.5 g/L peptone, 1 g/L yeast extract, 20 g/L agar and 33 g/L artificial sea salt [22], and sub-cultured every 20–30 days.

### 4.2. Sequencing and Phylogenetic Analysis

The isolated Labyrinthulomycete strains were identified based on their 18S rRNA gene sequences. Each of the isolated strains was cultured in M4 lipid medium (20 g/L glucose, 1.5 g/L peptone, 1 g/L yeast extract, 0.25 g/L KH_2_PO_4_ and 33 g/L artificial seawater) at 28 °C and 150 rpm for 4 days [23]. The resulting cell culture was centrifuged and washed twice with sterile artificial seawater to remove the medium constituents. Total genomic DNA was extracted from the washed cell pellet using a DNA extraction kit (Generay, Shanghai, China) following the manufacturer’s instructions. Three pairs of primers namely 18S001/18S13 [35], ThrMisF/ThrMisR [58], and Laby-A/Laby-Y [59] were employed for DNA amplification from the strains (Table S1). For each set of primers, a specific program (Table S2) was used for polymerase chain reaction (PCR) in an S1000™ thermal cycler (Bio-Rad, Hercules, CA, USA). The PCR product was checked by agarose gel electrophoresis and examined under a UV transilluminator. DNA products with two or more bands were cut from the gel and purified using a gel DNA extraction kit (Generay, Shanghai, China), and those with a single band were recovered directly using the PCR product recovery kit (Tiangen, Beijing, China). Two restriction enzymes, Hpa I and Sac II (Takara, Dalian, Liaoning, China), were used to distinguish the PCR products by comparing their restriction fragments. The selected PCR products were then ligated into pTOPO-T Vector (Gene-better^TM^, Beijing, China) and transformed into *Escherichia coli* cells (DH5α). Plasmid DNA in the white colonies (presumed positive) was extracted using a plasmid extraction kit (Tiangen, Beijing, China) to confirm the presence of the target DNA fragment by agarose gel electrophoresis. Positive clones were sequenced using M13 universal primers by Beijing Genomics Institute (BGI), China. The resultant sequences were compared with reference sequences in the National Center for Biotechnology Information (NCBI) database. The sequences were aligned with reference sequences retrieved from the NCBI database using the MAFFT program (https://www.ebi.ac.uk/Tools/msa/mafft/) and were subsequently used for the construction of a maximum-likelihood (ML) phylogenetic tree with FastTree2 [60]. *Bacillaria cf. paxillifer* BA14c and *Chrysophyceae* sp. CCMP261 were chosen as outgroups for the phylogenetic tree. All the sequences generated in this study have been submitted to GenBank and their accession numbers are provided in Table S1. 

### 4.3. Screening for DHA-Producing Strains

Seed culture was prepared according to the method described in section “Sample collection and strain isolation”. It was incubated in 100 mL flasks containing 50 mL M4 medium [42] at 28 °C and 150 rpm for 24 h. A 5 mL of seed broth was transferred to 50 mL fresh M4 medium and incubated for 4 days under the same conditions as that used for the seed culture. Two samples were collected from each culture, one of which was used for the primary screening by the Nile Red staining method to select the high lipid-accumulating strains. The other sample was used for the analysis of DHA titer of the selected samples by the gas chromatography (GC) method [23]. 

### 4.4. Culture Optimization for the Top Strain ZJWZ-7

Based on the screening results of DHA-producing strains, the top strain ZJWZ-7 was chosen for culture optimization. The medium nutrition (carbon, nitrogen, and KH_2_PO_4_) and environmental factors (salinity, initial pH, and rotation rate) were tested to evaluate their effects on the cell growth, DHA and SFAs production of the highest DHA-yielding strain ZJWZ-7 (Table S3). The OFAT experimental design and culture conditions for optimization are shown in Table S3. For all sets of experiments, the seed culture medium and conditions were the same as that used in the section “Screening for high DHA-producing strains”. The strain was incubated at 28 °C and 150 rpm for 4 days. All experiments were carried out in triplicates, and the results are expressed as means of the replicates with standard deviation (±SD).

In order to validate the cell growth and DHA production of ZJWZ-7 obtained under the optimal conditions derived from OFAT experiments, a further batch experiment was conducted. The experiment was performed in 500 mL flasks with a working volume of 300 mL containing the optimal medium. On the fourth day of the batch culture, samples were collected for the measurement of biomass as dry cell weight (DCW), DHA, and SFAs. The experiment was carried out in triplicate and the results are expressed as mean with standard deviation (±SD). 

### 4.5. Analytical Methods

The cell biomass (DCW) was determined by the gravimetric method. Briefly, an aliquot of culture broth was extracted to centrifugal tubes (pre-dried to constant weight) and centrifuged at 10,000 rpm for 5 min. The resulting cells were washed twice with sterile distilled water, lyophilized for 48 h using a freeze-dryer (Christ, Gefriertrocknungsanlagen, Osterode am Harz, Germany), and weighed. The cell pellets were stored at −80 °C for further fatty acids analysis [23].

Nile Red staining was performed by adding 0.01% (w/v) Nile Red-acetone solution to the cell pellet and incubating for ca. 1 min before observation under an Epifluorescence Microscope (Nikon DS-Ri1) at the wavelength of 540 nm. Strains with high intracellular lipid accumulation exhibiting high intensity of fluorescence were selected for further analysis of the composition of their fatty acids.

Analysis of fatty acid composition was carried out using the direct transesterification method [61]. In brief, freeze-dried cells (50–100 mg) were transferred to 2 mL of 4% sulfuric acid in methanol, with the addition of 100 μL of nonadecanoic acid (1.0 mg/mL) solution (Sigma-Aldrich, St. Louis, MO, USA) as an internal standard. The mixture was vortexed for 30 s, then incubated at 80 °C for 1 h for methyl-esterification. After the mixture was cooled down to room temperature, 1 mL each of distilled water and n-hexane was added to it. The fatty acid methyl esters (FAMEs) of the mixture were dissolved in the hexane layer after they were vortexed and centrifuged at 4000 rpm for 5 min. The FAMEs were collected and analyzed by gas chromatography Agilent 7890B (Agilent, Santa Clara, CA, USA) equipped with a DB-WAX column (60 m × 320 μm × 0.15 μm) and flame ionization detector (FID). Samples were injected in the split injection mode with the split ratio of 50:1. The injection port temperature was set to 250 °C. Nitrogen was used as the carrier gas with a flow of 1 mL/min. The column temperature was originally set to 50 °C for 1 min, followed by programming at 25 °C/min to 175 °C, then increased to 220 °C at 3 °C/min and held for 5 min, finally reached to 230 °C at 2 °C/min and held for 11 min.

### 4.6. Statistical Analysis

The significant effects of various factors (carbon and nitrogen sources, pH, salinity, temperature, and rotation rate) on cell biomass, DHA, and SFAs were tested by one-way analysis of variance (ANOVA) at alpha level 0.05 using SPSS Statistic 19 software. 

## 5. Conclusions

A total of 71 thraustochytrid and labyrinthulid strains belonging to six different genera were isolated and identified from different coastal mangroves of China. Phylogenetic analysis of these strains revealed a monophyletic relationship of thraustochytrid and labyrinthulid groups. Further screening of 13 thraustochytrid and two labyrinthulid strains demonstrated their high DHA production potential. One strain, ZJWZ-7, exhibited the maximum production of DHA (1.66 g/L, 44.52% of TFAs) and SFAs (1.68 g/L, 44.40% of TFAs) under optimal culture conditions. This culture-based study expands the current catalogue of Labyrinthulomycete protists and provides the molecular phylogeny and lipid production profiles of several novel thraustochytrid and labyrinthulid strains.

## Figures and Tables

**Table 1:** Information about sampling, isolation and identification of thraustochytrid and labyrinthulid strains.

Sampling Site	Sampling Date	Latitude (°N)	Longitude (°E)	Number of Isolates	Genus Level Classification	Number of Strains
Haikou, Hainan (HNHK)	2015.07	20.06231	110.3352	69	*Aurantiochytrium, Schizochytrium, Thraustochytrium, Botryochytrium, Parietichytrium, Thraustochytriidae, Labyrinthula*	17
Haikou, Hainan (HNU)	2016.03	20.06344	110.3345	23	*Aurantiochytrium, Labyrinthula*	6
Beihai, Guangxi (GXBH)	2016.04	21.41175	109.1626	54	*Aurantiochytrium, Schizochytrium, Thraustochytrium, Thraustochytriidae, Labyrinthula*	32
Qinzhou, Guangxi (GXQZ)	2016.04	21.74423	108.5903	16	*Aurantiochytrium, Thraustochytrium, Labyrinthula*	11
Shenzhen, Guangdong (GDSZ)	2015.08	22.52029	113.9496	22	*Thraustochytrium, Thraustochytriidae*	2
Longhai, Fujian (FJLH)	2015.09	24.43823	117.9037	7	*Aurantiochytrium*	2
Quanzhou, Fujian (FJQZ)	2015.09	24.94337	118.6748	6	*Aurantiochytrium*	0
Zhangzhou, Fujian (FJZZ)	2015.09	23.92075	117.4167	10	*Aurantiochytrium*, *Schizochytrium*, *Thraustochytrium,*	0
Wenzhou, Zhejiang (ZJWZ)	2015.09	28.34687	121.1738	5	*Aurantiochytrium*	1

**Table 2:** List of different genera of thraustochytrid and labyrinthulid strains isolated in this study.

Group	Genus	Number of Isolates in Each Genus	Number of Sequences Submitted
Thraustochytrids	*Aurantiochytrium*	129	27
	*Schizochytrium*	46	7
	*Thraustochytrium*	6	6
	*Botryochytrium*	1	1
	*Parietichytrium*	2	2
Labyrinthulids	*Labyrinthula*	28	28

**Table 3:** Fatty acid composition (percentage), biomass, and yield and productivity of total fatty acids (TFAs) of high docosahexaenoic acid (DHA)-producing (>10% biomass) thraustochytrid and labyrinthulid strains.

**Strain**	**ZJWZ-7**	**HNHK-100**	**GXBH-216**	**GXBH-227**	**GXBH-220**	**GXBH-228**	**GXBH-232**	**GXBH-110**
C12:0	1.49 ± 0.26	1.45 ± 0.12	0.56 ± 0.08	0.10 ± 0.02	0.96 ± 0.10	0.43 ± 0.22	1.13 ± 0.10	0.70 ± 0.27
C14:0	4.85 ± 0.02	4.35 ± 0.04	3.90 ± 0.06	4.10 ± 0.34	4.70 ± 0.04	5.01 ± 0.12	4.39 ± 0.09	5.01 ± 0.04
C15:0	1.88 ± 0.01	1.74 ± 0.01	1.75 ± 0.18	4.58 ± 0.27	2.09 ± 0.00	2.01 ± 0.00	2.19 ± 0.04	1.82 ± 0.02
C16:0	41.77 ± 0.33	46.08 ± 0.29	37.19 ± 0.63	46.32 ± 1.02	36.11 ± 0.33	40.09 ± 0.23	36.70 ± 0.37	42.57± 0.14
C17:0	0.48 ± 0.00	0.53 ± 0.00	0.66 ± 0.07	1.40 ± 0.03	0.51 ± 0.01	0.49 ± 0.00	0.57 ± 0.01	0.44 ± 0.01
C18:0	0.91 ± 0.02	1.09 ± 0.00	0.88 ± 0.04	0.95 ± 0.04	0.73 ± 0.04	0.76 ± 0.00	0.83 ± 0.01	0.80 ± 0.03
C18:1n9	0.38 ± 0.00	0.32 ± 0.00	0.54 ± 0.10	0.11 ± 0.00	0.43 ± 0.00	0.49 ± 0.00	0.46 ± 0.00	0.33 ± 0.00
C20:4n6	0.44 ± 0.00	0.39 ± 0.00	0.50 ± 0.02	0.02 ± 0.03	0.55 ± 0.00	0.53 ± 0.01	0.53 ± 0.01	0.49 ± 0.00
C20:5n3	0.32 ± 0.00	0.24 ± 0.00	0.48 ± 0.06	0.26 ± 0.02	0.38 ± 0.01	0.37 ± 0.00	0.34 ± 0.01	0.35 ± 0.02
C22:5n3	7.63 ± 0.01	7.42 ± 0.02	9.77 ± 0.27	8.23 ± 0.40	8.52 ± 0.06	7.98 ± 0.01	9.26 ± 0.17	7.48 ± 0.09
C22:6n3	37.68 ± 0.06	34.31 ± 0.17	42.95 ± 1.02	28.54 ± 1.34	42.33 ± 0.54	39.60 ± 0.27	41.24 ± 0.35	37.72± 0.28
Total SFAs	51.38	55.25	44.94	57.45	45.11	48.79	45.8	51.42
Total PUFAs	46.07	42.36	53.69	37.05	51.77	48.47	51.38	46.04
Biomass (g/L)	6.83 ± 0.11	6.72 ± 0.22	1.72 ± 0.22	4.85 ± 0.45	7.52 ± 0.06	7.28 ± 0.23	7.45 ± 0.11	7.28 ± 0.07
TFAs yield (% biomass)	44.18 ± 1.15	40.2 ± 2.24	32.03 ± 5.88	47.57 ± 0.36	30.69 ± 0.50	32.57 ± 0.21	30.68 ± 7.09	33.5 ± 0.47
TFAs productivity (g/L/d)	0.74 ± 0.01	0.67 ± 0.02	0.14 ± 0.01	0.58 ± 0.03	0.57 ± 0.01	0.58 ± 0.02	0.56 ± 0.14	0.60 ± 0.01
**Strain**	**GXBH-111**	**GXBH-114**	**GXBH-137**	**GXBH-135**	**GXBH-233**	**GXBH-107**	**GXBH-215**	
C12:0	0.12 ± 0.02	0.66 ± 0.10	0.14 ± 0.03	0.13 ± 0.04	0.15 ± 0.02	0.62 ± 0.25	0.12 ± 0.01	
C14:0	4.33 ± 0.51	5.70 ± 0.04	3.89 ± 0.16	4.67 ± 0.39	3.87 ± 0.19	4.56 ± 0.02	4.50 ± 0.22	
C15:0	2.16 ± 0.13	1.72 ± 0.01	2.16 ± 0.09	1.73 ± 0.09	1.86 ± 0.04	1.54 ± 0.01	1.38 ± 0.02	
C16:0	40.61 ± 0.67	42.16 ± 0.04	38.54 ± 1.71	46.38 ± 0.49	42.47 ± 0.29	37.03 ± 0.07	45.89 ± 0.51	
C17:0	0.71 ± 0.02	0.44 ± 0.00	0.64 ± 0.11	0.56 ± 0.11	0.71 ± 0.01	0.38 ± 0.00	0.38 ± 0.01	
C18:0	0.87 ± 0.06	0.77 ± 0.00	0.88 ± 0.03	0.91 ± 0.06	0.96 ± 0.03	0.71 ± 0.00	1.08 ± 0.09	
C18:1n9	0.45 ± 0.09	0.39 ± 0.00	0.37 ± 0.03	0.38 ± 0.02	0.40 ± 0.01	0.46 ± 0.00	0.38 ± 0.00	
C20:4n6	0.51 ± 0.02	0.48 ± 0.01	0.68 ± 0.29	0.42 ± 0.01	0.43 ± 0.01	0.58 ± 0.01	0.41 ± 0.01	
C20:5n3	0.32 ± 0.03	0.39 ± 0.00	0.33 ± 0.02	0.45 ± 0.04	0.50 ± 0.02	0.40 ± 0.00	0.49 ± 0.01	
C22:5n3	8.96 ± 0.50	7.31 ± 0.06	8.81 ± 0.42	7.77 ± 0.15	8.42 ± 0.28	8.36 ± 0.04	7.82 ± 0.04	
C22:6n3	39.55 ± 0.89	37.80 ± 0.16	39.38 ± 2.72	35.54 ± 0.30	39.14 ± 0.30	42.73 ± 0.09	36.32 ± 0.32	
Total SFAs	48.81	51.44	46.25	54.38	50.03	44.85	53.35	
Total PUFAs	49.34	45.98	49.21	44.17	48.48	52.07	45.04	
Biomass (g/L)	6.48 ± 0.25	7.22 ± 0.10	6.97 ± 0.18	6.72 ± 0.18	7.00 ± 0.06	7.51 ± 0.26	6.8 ± 0.32	
TFAs yield (% biomass)	31.6 ± 0.98	30.26 ± 0.23	28.67 ± 4.18	31.48 ± 4.32	26.07 ± 1.90	30.88 ± 2.64	30.4 ± 0.51	
TFAs productivity (g/L/d)	0.51 ± 0.02	0.54 ± 0.00	0.50 ± 0.06	0.53 ± 0.24	0.46 ± 0.03	0.57 ± 0.03	0.52 ± 0.03	

Note: SFAs: saturated fatty acids; PUFAs: polyunsaturated fatty acids.

**Table 4:** Significant (*P* < 0.05, analysis of variance (ANOVA)) parameters that influenced the cell biomass, DHA, and SFAs production of *Aurantiochytrium* sp. ZJWZ-7.

Cell biomass (DCW, g/L)	DHA (g/L)	SFAs (g/L)
Carbon source	Carbon source	Nitrogen source
Nitrogen source	Nitrogen source	KH_2_PO_4_
Salinity	Initial pH	Salinity
Initial pH	Temperature	Initial pH
Temperature	Rotation rate	Temperature
Rotation rate		Rotation rate

**Table 5:** Effect of medium components and process conditions on the cell biomass, DHA and SFAs production of *Aurantiochytrium* sp. ZJWZ-7.

Culture Condition	Biomass (g/L)	DHA (g/L)	SFAs (g/L)	DHA (% TFAs)	SFAs (% TFAs)
KH_2_PO_4_ (g/L)					
0	6.53	1.20	1.69	36.70	51.34
0.1	6.81	1.31	1.85	37.56	51.72
0.25	6.83	1.14	1.52	37.68	50.36
0.4	6.82	1.14	1.53	37.94	49.72
0.8	6.99	1.07	1.45	37.62	49.78
Salinity (% seawater)					
0	5.62	0.91	1.05	35.32	51.44
20	6.99	0.99	1.04	37.32	49.28
40	6.87	0.87	0.95	37.21	50.53
60	6.88	1.10	1.25	36.43	51.96
80	6.74	0.90	0.97	36.95	50.26
100	6.83	1.14	1.52	37.68	50.36
120	6.61	0.90	0.94	36.87	48.65
Temperature (℃)					
20	6.56	1.01	1.58	39.41	50.46
25	6.58	1.14	1.49	38.38	50.76
28	6.83	1.14	1.52	37.68	50.36
32	5.73	0.91	1.29	35.86	50.08
35	4.98	0.51	0.83	34.01	53.82
Initial pH					
4	7.21	1.21	1.39	40.62	46.09
5	6.57	1.04	1.53	36.33	52.76
6	6.46	1.07	1.33	37.87	50.49
6.47	6.83	1.14	1.52	37.68	50.36
7	6.54	1.23	1.41	40.68	46.51
8	5.49	0.82	1.23	35.08	52.80
Rotation rate (rpm)					
100	6.14	0.91	1.08	39.91	47.86
150	6.83	1.14	1.52	37.68	50.36
180	6.41	1.04	1.58	33.88	53.95
200	6.51	0.99	1.33	36.67	50.57
250	3.55	0.58	1.21	35.46	53.37

**Table 6:** Fatty acid composition (% TFAs) of 4-days grown *Aurantiochytrium* sp. ZJWZ-7 culture on optimal medium.

Strains	ZJWZ-7
C12:0	0.15 ± 0.00
C14:0	3.28 ± 0.02
C15:0	1.89 ± 0.02
C16:0	37.63 ± 0.15
C17:0	0.22 ± 0.00
C18:0	0.84 ± 0.02
C18:3n3	0.17 ± 0.00
C20:0	0.12 ± 0.00
C21:0	0.27 ± 0.01
C20:3n6	0.42 ± 0.03
C20:4n6	0.23 ± 0.00
C20:5n3	0.06 ± 0.00
C22:5n3	7.57 ± 0.00
C22:6n3	44.52 ± 0.12
Total PUFAs	52.97
Total SFAs	44.40
Biomass (g/L)	9.88
TFAs yield (% biomass)	37.69
DHA (g/g)	0.17
DHA (g/L)	1.66
PUFAs (g/L)	2.00
SFAs (g/g)	0.17
SFAs (g/L)	1.68

Note: PUFAs: polyunsaturated fatty acids; SFAs: saturated fatty acids; TFAs: total fatty acids; DHA: docosahexaenoic acid.

## Supplementary Materials

The following are available online at https://www.mdpi.com/1660-3397/17/5/268/s1, Figure S1: Microscope images (a-i) and fluorescence micrographs (j-m) of the thraustochytrids and labyrinthulids. Thraustochytrids: a-h, j, k; labyrinthulids: i, l, m., Figure S2: Cell biomass, DHA and SFA production of Aurantiochytrium sp. ZJWZ-7 at different growth stages., Table S1: Information about 71 Labyrinthulomycete strains isolated from mangroves of China in this study., Table S2: Primer-specific PCR programs employed for DNA amplification from isolated strains., Table S3: One-factor-at-a-time (OFAT) experimental design for optimization of DHA production in strain ZJWZ-7.

## References

[1] Bennett R.M., Honda D., Beakes G.W., Thines M., Archibald J.M., Simpson A.G.B., Slamovits C.H., Margulis L., Melkonian M., Chapman D.J., Corliss J.O. (2017). Labyrinthulomycota. Handbook of the Protists.

[2] Leander C.A., Porter D. (2000). Redefining the genus Aplanochytrium (phylum Labyrinthulomycota). Mycotaxon.

[3] Leander C.A., Porter D. (2001). The Labyrinthulomycota Is Comprised of Three Distinct Lineages. Mycologia.

[4] Leander C.A., Porter D., Leander B.S. (2004). Comparative morphology and molecular phylogeny of aplanochytrids (Labyrinthulomycota). Eur. J. Protistology.

[5] Raghukumar S. (2002). Ecology of the marine protists, the Labyrinthulomycetes (thraustochytrids and labyrinthulids). Eur. J. Protistology.

[6] Bremer G.B. (1995). Lower marine fungi (Labyrinthulomycetes) and the decay of mangrove leaf litter. Hydrobiologia.

[7] Singh P., Liu Y., Li L., Wang G. (2014). Ecological dynamics and biotechnological implications of thraustochytrids from marine habitats. Appl. Microbiol. Biotechnol..

[8] Raghukumar S., Ramaiah N. (2004). The Role of Fungi in Marine Detrital Processes. Marine Microbiology: Facets and Opportunities.

[9] Raghukumar S., Damare V.S. (2011). Increasing evidence for the important role of Labyrinthulomycetes in marine ecosystems. Bot. Mar..

[10] Raghukumar S. (2017). Fungi in Coastal and Oceanic Marine Ecosystems.

[11] Hinzpeter I., Quilodrán B., Stead R., Trujillo L., Vidal J., Shene C. (2009). Isolation of thraustochytrid strains in the coastal zone of Puerto Montt, Chile and evaluation of Docosahexaenoic acid (22:6n-3, DHA) production. Afinidad.

[12] Gupta A., Wilkens S., Adcock J.L., Puri M., Barrow C.J. (2013). Pollen baiting facilitates the isolation of marine thraustochytrids with potential in omega-3 and biodiesel production. J. Ind. Microbiol. Biotechnol..

[13] Caamaño E., Loperena L., Hinzpeter I., Pradel P., Gordillo F., Corsini G., Tello M., Lavín P., González A.R. (2017). Isolation and molecular characterization of Thraustochytrium strain isolated from Antarctic Peninsula and its biotechnological potential in the production of fatty acids. Brazilian J. Microbiol..

[14] Unagul P., Suetrong S., Preedanon S., Klaysuban A., Gundool W., Suriyachadkun C., Sakayaroj J. (2017). Isolation, fatty acid profiles and cryopreservation of marine thraustochytrids from mangrove habitats in Thailand. Bot. Mar..

[15] Sullivan B.K., Robinson K.L., Trevathan-Tackett S.M., Lilje E.S., Gleason F.H., Lilje O. (2017). The First Isolation and Characterisation of the Protist Labyrinthula sp. in Southeastern Australia. J. Eukaryotic Microbiol..

[16] Boro M.C., Harakava R., Pires-Zottarelli C.L.A. (2018). Labyrinthulomycota from Brazilian mangrove swamps and coastal waters. Botanica Marina.

[17] Ueda M., Nomura Y., Doi K., Nakajima M., Honda D. (2015). Seasonal dynamics of culturable thraustochytrids (Labyrinthulomycetes, Stramenopiles) in estuarine and coastal waters. Aquat. Microb. Ecol..

[18] Lee Chang K.J., Dunstan G.A., Abell G.C.J., Clementson L.A., Blackburn S.I., Nichols P.D., Koutoulis A. (2012). Biodiscovery of new Australian thraustochytrids for production of biodiesel and long-chain omega-3 oils. Appl. Microbiol. Biotechnol..

[19] Burja A.M., Radianingtyas H., Windust A., Barrow C.J. (2006). Isolation and characterization of polyunsaturated fatty acid producing Thraustochytrium species: Screening of strains and optimization of omega-3 production. Appl. Microbiol. Biotechnol..

[20] Damare V.S. (2015). Diversity of thraustochytrid protists isolated from brown alga, *Sargassum cinereum* using 18S rDNA sequencing and their morphological response to heavy metals. J. Mar. Biol. Assoc. U. K..

[21] Jaseera K.V., Kaladharan P., Vijayan K.K., Sandhya S.V., Antony M.L., Pradeep M.A. (2018). Isolation and phylogenetic identification of heterotrophic thraustochytrids from mangrove habitats along the southwest coast of India and prospecting their PUFA accumulation. J. Appl. Phycol..

[22] Damare V., Raghukumar S. (2006). Morphology and Physiology of the Marine Straminipilan Fungi, the Aplanochytrids Isolated from the Equatorial Indian Ocean. Indian J. Mar. Sci..

[23] Liu Y., Singh P., Sun Y., Luan S., Wang G. (2014). Culturable diversity and biochemical features of thraustochytrids from coastal waters of Southern China. Appl. Microbiol. Biotechnol..

[24] Hong W.K., Rairakhwada D., Seo P.S., Park S.Y., Hur B.K., Kim C.H., Seo J.W. (2011). Production of lipids containing high levels of docosahexaenoic acid by a newly isolated microalga, *Aurantiochytrium* sp. KRS101. Appl. Microbiol. Biotechnol..

[25] Manikan V., Nazir M.Y.M., Kalil M.S., Isa M.H.M., Kader A.J.A., Yusoff W.M.W., Hamid A.A. (2015). A new strain of docosahexaenoic acid producing microalga from Malaysian coastal waters. Algal Res..

[26] Jaritkhuan S., Suanjit S. (2018). Species diversity and polyunsaturated fatty acid content of thraustochytrids from fallen mangrove leaves in Chon Buri province, Thailand. Agrc. Nat. Resour..

[27] Kumon Y., Yokoyama R., Haque Z., Yokochi T., Honda D., Nakahara T. (2006). A New Labyrinthulid Isolate That Produces Only Docosahexaenoic Acid. Mar. Biotechnol..

[28] Aasen I.M., Ertesvåg H., Heggeset T.M.B., Liu B., Brautaset T., Vadstein O., Ellingsen T.E. (2016). Thraustochytrids as production organisms for docosahexaenoic acid (DHA), squalene, and carotenoids. Appl. Microbiol. Biotechnol..

[29] Huang J., Aki T., Yokochi T., Nakahara T., Honda D., Kawamoto S., Shigeta S., Ono K., Suzuki O. (2003). Grouping Newly Isolated Docosahexaenoic Acid-Producing Thraustochytrids Based on Their Polyunsaturated Fatty Acid Profiles and Comparative Analysis of 18S rRNA Genes. Mar. Biotechnol..

[30] Jakobsen A.N., Aasen I.M., Josefsen K.D., Strøm A.R. (2008). Accumulation of Docosahexaenoic Acid-Rich Lipid in Thraustochytrid *Aurantiochytrium* sp. strain T66: Effects of N and P Starvation and O_2_ Limitation. Appl. Microbiol. Biotechnol..

[31] Chaung K.C., Chu C.Y., Su Y.M., Chen Y.M. (2012). Effect of culture conditions on growth, lipid content, and fatty acid composition of *Aurantiochytrium mangrovei* strain BL10. AMB Express.

[32] Qu L., Ren L.J., Sun G.N., Ji X.J., Nie Z.K., Huang H. (2013). Batch, fed-batch and repeated fed-batch fermentation processes of the marine thraustochytrid schizochytrium sp. For producing docosahexaenoic acid. Bioprocess. Biosyst. Eng..

[33] Wang Q., Ye H., Sen B., Xie Y., He Y., Park S., Wang G. (2018). Improved production of docosahexaenoic acid in batch fermentation by newly-isolated strains of *Schizochytrium* sp. and Thraustochytriidae sp. through bioprocess optimization. Synth. Syst. Biotechnol..

[34] Wang Q., Sen B., Liu X., He Y., Xie Y., Wang G. (2018). Enhanced saturated fatty acids accumulation in cultures of newly-isolated strains of *Schizochytrium* sp. and Thraustochytriidae sp. for large-scale biodiesel production. Sci. Total Environ..

[35] Honda D., Yokochi T., Nakahara T., Raghukumar S., Nakagiri A., Schaumann K., Higashihara T. (1999). Molecular Phylogeny of Labyrinthulids and Thraustochytrids Based on the Sequencing of 18S Ribosomal RNA Gene. J. Eukaryotic Microbiol..

[36] Honda D., Yokochi T., Nakahara T., Erata M., Higashihara T. (1998). *Schizochytrium limacinum* sp. nov., a new thraustochytrid from a mangrove area in the west Pacific Ocean. Mycol. Res..

[37] Fan K.W., Vrijmoed L.L.P., Jones E.B.G. (2002). Physiological studies of subtropical mangrove thraustochytrids. Bot. Mar..

[38] Rosa S.M., Galvagno M.A., Ve’lez C.G. (2011). Adjusting culture conditions to isolate thraustochytrids from temperate and cold environments in southern Argentina. Mycoscience.

[39] Fuller M.S., Jaworski A. (1987). Zoosporic fungi in teaching and research. Mycologia.

[40] Li Q., Chen G.Q., Fan K.W., Lu F.P., Aki T., Jiang Y. (2009). Screening and characterization of squalene-producing thraustochytrids from Hong Kong mangroves. J. Agric. Food. Chem..

[41] Gupta A., Singh D., Byreddy A.R., Thyagarajan T., Sonkar S.P., Mathur A.S., Tuli D.K., Barrow C.J., Puri M. (2016). Exploring omega-3 fatty acids, enzymes and biodiesel producing thraustochytrids from Australian and Indian marine biodiversity. Biotechnol. J..

[42] Jain R., Raghukumar S., Sambaiah K., Kumon Y., Nakahara T. (2007). Docosahexaenoic acid accumulation in thraustochytrids: Search for the rationale. Mar. Biol..

[43] Perveen Z., Ando H., Ueno A., Ito Y., Yamamoto Y., Yamada Y., Takagi T., Kaneko T., Kogame K., Okuyama H. (2006). Isolation and characterization of a novel thraustochytrid-like microorganism that efficiently produces docosahexaenoic acid. Biotechnol. Lett..

[44] Marchan L.F., Lee Chang K.J., Nichols P.D., Polglase J.L., Mitchell W.J., Gutierrez T. (2017). Screening of new British thraustochytrids isolates for docosahexaenoic acid (DHA) production. J. Appl. Phycol..

[45] Erwin J. (1973). Comparative biochemistry of fatty acids in eukaryotic microorganisms. Lipids Biomembr. Eukaryot. Microorg..

[46] Yaguchi T., Tanaka S., Yokochi T., Nakahara T., Higashihara T. (1997). Production of High Yields of Docosahexaenoic Acid by *Schizochytrium* sp. Strain SR21. J. Am. Oil Chemists’ Soc..

[47] Zeng Y., Ji X.J., Lian M., Ren L.J., Jin L.J., Ouyang P.K., Huang H. (2011). Development of a temperature shift strategy for efficient docosahexaenoic acid production by a marine fungoid protist, *Schizochytrium* sp. HX-308. Appl. Biochem. Biotechnol..

[48] Huang T.Y., Lu W.C., Chu I.M. (2012). A fermentation strategy for producing docosahexaenoic acid in *Aurantiochytrium limacinum* SR21 and increasing C22:6 proportions in total fatty acid. Bioresour. Technol..

[49] Sun X.M., Ren L.J., Ji X.J., Chen S.L., Guo D.S., Huang H. (2016). Adaptive evolution of *Schizochytrium* sp. by continuous high oxygen stimulations to enhance docosahexaenoic acid synthesis. Bioresour. Technol..

[50] Ralph P.J., Short F.T. (2002). Impact of the wasting disease pathogen, *Labyrinthula zosterae*, on the photobiology of eelgrass Zostera marina. Mar. Ecol. Prog. Ser..

[51] Jakobsson-Thor S., Toth G.B., Brakel J., Bockelmann A.C., Pavia H. (2018). Seagrass wasting disease varies with salinity and depth in natural Zostera marina populations. Mar. Ecol. Prog. Ser..

[52] Raghukumar S. (2008). Thraustochytrid marine protists: Production of PUFAs and other emerging technologies. Mar. Biotechnol..

[53] Bowles R.D., Hunt A.E., Bremer G.B., Duchars M.G., Eaton R.A. (1999). Long-chain n-3 polyunsaturated fatty acid production by members of the marine protistan group the thraustochytrids: Screening of isolates and optimization of docosahexaenoic acid production. J. Biotechnol..

[54] Wu S.-T., Yu S.-T., Lin L.-P. (2005). Effect of culture conditions on docosahexaenoic acid production by Schizochytrium sp. S31. Process Biochem..

[55] Scott S.D., Armenta R.E., Berryman K.T., Norman A.W. (2011). Use of raw glycerol to produce oil rich in polyunsaturated fatty acids by a thraustochytrid. Enzyme Microb. Technol..

[56] Quilodrán B., Hinzpeter I., Quiroz A., Shene C. (2009). Evaluation of liquid residues from beer and potato processing for the production of docosahexaenoic acid (C22:6n-3, DHA) by native thraustochytrid strains. World J. Microbiol. Biotechnol..

[57] Liang Y., Sarkany N., Cui Y., Yesuf J., Trushenski J., Blackburn J.W. (2010). Use of sweet sorghum juice for lipid production by *Schizochytrium limacinum* SR21. Bioresour. Technol..

[58] Bai M., Sen B., Wang Q., Xie Y., He Y., Wang G. (2018). Molecular Detection and Spatiotemporal Characterization of Labyrinthulomycete Protist Diversity in the Coastal Waters Along the Pearl River Delta. Microb. Ecol..

[59] Collado-Mercado E., Radway J.C., Collier J.L. (2010). Novel uncultivated labyrinthulomycetes revealed by 18S rDNA sequences from seawater and sediment samples. Aquat. Microb. Ecol..

[60] Price M.N., Dehal P.S., Arkin A.P. (2010). FastTree 2—Approximately Maximum-Likelihood Trees for Large Alignments. PLOS ONE.

[61] Lepage G., Roy C.C. (1984). Improved recovery of fatty acid through direct transesterification without prior extraction or purification. J. Lipid Res..

