# Supplementary material for: Culturable Diversity and Lipid Production Profile of Labyrinthulomycete Protists Isolated from Coastal Mangrove Habitats of China
Source: Mar Drugs. 2019 May 6;17(5):268. doi: 10.3390/md17050268 (PMC6562557; doi:10.3390/md17050268)
Supplement: Supplementary file 1 [file marinedrugs-17-00268-s001.pdf]

Supplementary Information

Culturable Diversity and Lipid Production Profile of Labyrinthulomycete Protists Isolated from Coastal Mangrove Habitats of China

Quizhen Wang[1,3,†], Huike Ye[1,†], Yunxuan Xie[1], Yaodong He[1], Biswarup Sen[1] and Guangyi Wang[1,2,*]

[1] Center for Marine Environmental Ecology, School of Environmental Science and Engineering, Tianjin University, Tianjin 300072, China; qqzz1990@tju.edu.cn (Q.W.); yehuike@163.com (H.Y.); yxie@tju.edu.cn (Y.X.), yaodong.he@tju.edu.cn (Y.H.); bsen@tju.edu.cn (B.S.); gywang@tju.edu.cn (G.W.)
[2] Key Laboratory of Systems Bioengineering (Ministry of Education), Tianjin University, Tianjin 300072, China gywang@tju.edu.cn (G.W.)
[3] Ocean College of Hebei Agricultural University, Qinhuangdao, 066000, China; 806756201@qq.com (Q.W.)
† These authors contributed equally to this work
* Correspondence: gywang@tju.edu.cn; Tel: (86) 022-8740210

*To whom correspondence should be addressed.

Email: gywang@tju.edu.cn

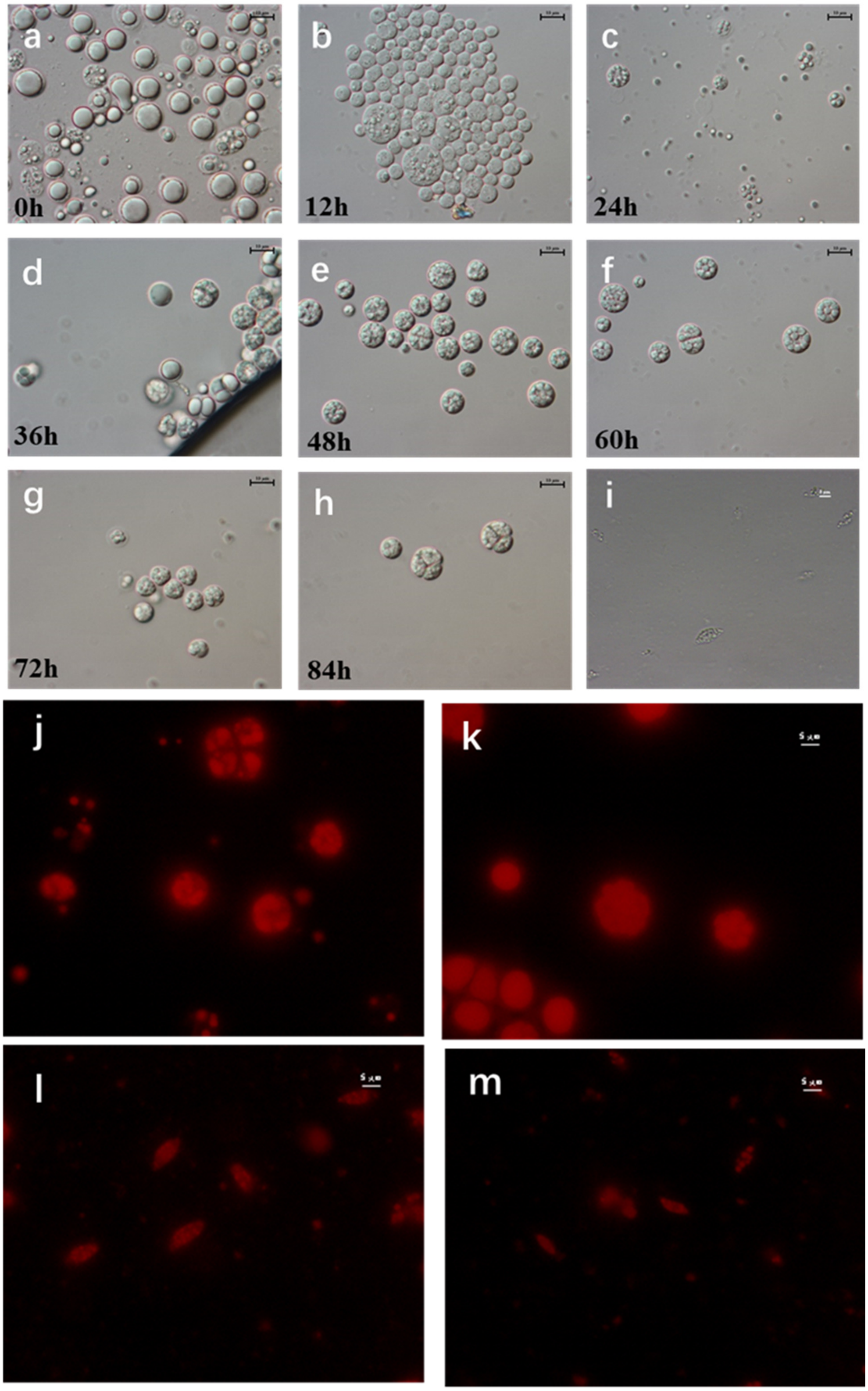

Fig. S1. Microscope images (a-i) and fluorescence micrographs (j-m) of the thraustochytrids and labyrinthulids isolated in the present study. Thraustochytrids: a-h, j, k; labyrinthulids: i, l, m.

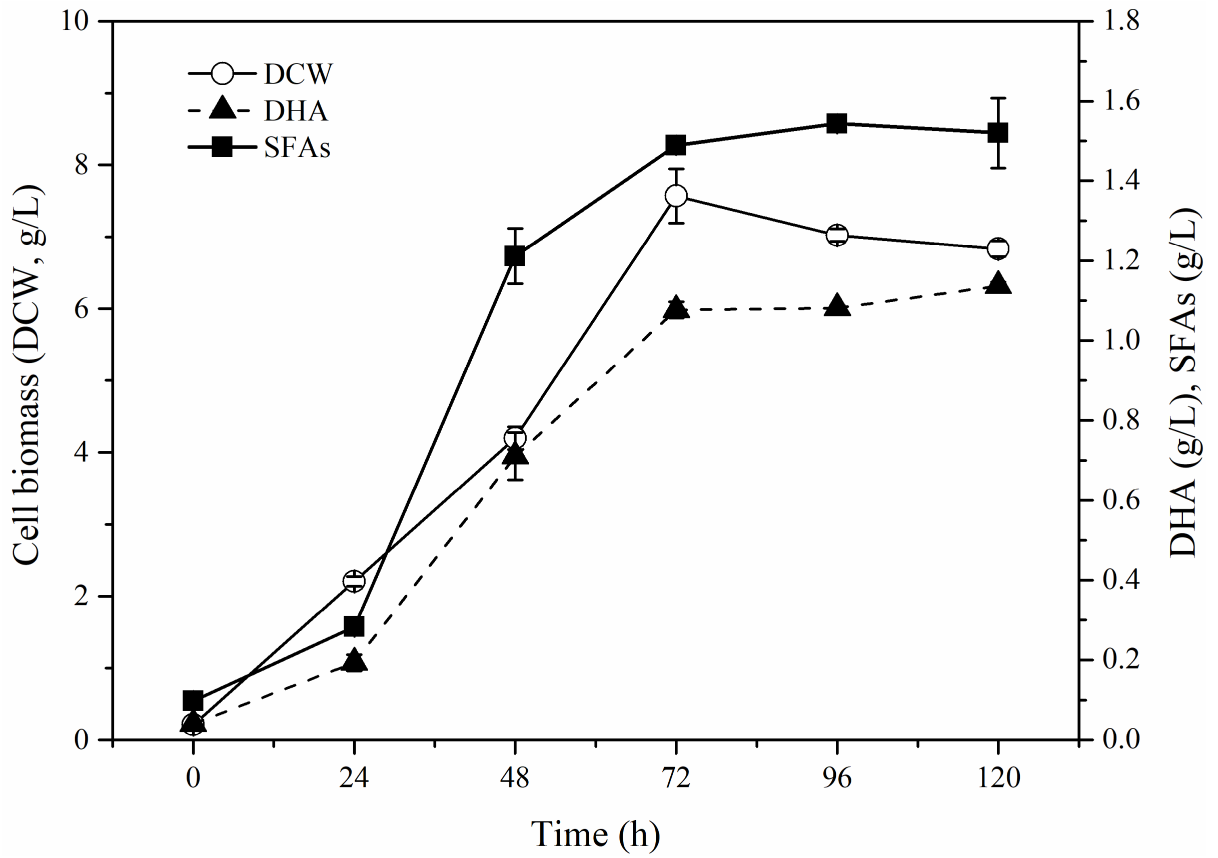

Fig. S2. Cell biomass, DHA and SFA production of *Aurantiochytrium* sp. ZJWZ-7 at different growth stages.

Table S1. Information about 71 Labyrinthulomycete strains isolated from mangroves of China in this study.

No.	Isolate	DHA (g/g)	DHA (g/l)	PCR Primers for identification	Closest relative (GenBank accession number)	Query coverage (%)	Identity (%)	Sampling site	Latitude (°N)	Longitude (°E)	GenBank accession number
1	ZJWZ-7	0.166	1.137	18S001 / 18S13	*Aurantiochytrium* sp. (KM023712.1)	100	100	Wenzhou, Zhejiang	28.346867	121.173817	MG429119.1
2	HNHK-100	0.138	0.926	18S001 /18S13	*Aurantiochytrium* sp. (FJ821477.1)	100	99	Haikou, Hainan	20.062314	110.335232	MG429118.1
3	GXBH-216	0.138	0.234	ThrMisF / ThrMisR	*Aurantiochytrium* sp. (AB900161.1)	100	99	Beihai, Guangxi	21.41175	109.162633	MG429110.1
4	GXBH-227	0.132	0.640	Laby A / LabyY	*Thraustochytrium* sp. (DQ374149.1)	94	95	Beihai, Guangxi	21.41175	109.162633	MG429091.1
5	GXBH-107	0.132	0.989	Laby A / LabyY	*Labyrinthula* sp. (AB246794.1)	100	100	Beihai, Guangxi	21.41175	109.162633	MG429087.1
6	GXBH-220	0.130	0.977	ThrMisF / ThrMisR	*Aurantiochytrium* sp. (KM023713.1)	100	99	Beihai, Guangxi	21.41175	109.162633	MG429111.1
7	GXBH-228	0.129	0.939	ThrMisF / ThrMisR	*Aurantiochytrium* sp. BL10 (FJ821477.1)	100	100	Beihai, Guangxi	21.41175	109.162633	MG429120.1
8	GXBH-232	0.127	0.945	ThrMisF / ThrMisR	*Aurantiochytrium* sp. (JQ982491.1)	100	100	Beihai, Guangxi	21.41175	109.162633	MG429114.1
9	GXBH-110	0.126	0.920	ThrMisF / ThrMisR	*Aurantiochytrium* sp. (KM023691.1)	100	100	Beihai, Guangxi	21.41175	109.162633	MG429124.1
10	GXBH-111	0.125	0.809	ThrMisF / ThrMisR	*Aurantiochytrium* sp. (JQ982488.1)	100	99	Beihai, Guangxi	21.41175	109.162633	MG429101.1
11	GXQZ-14	0.114	0.826	ThrMisF / ThrMisR	*Aurantiochytrium* sp. (AB810944.1)	100	100	Qinzhou, Guangxi	21.744233	108.59025	MG429116.1

12	GXBH-137	0.112	0.783	ThrMisF / ThrMisR	*Schizochytrium* sp. (JX847364.1)	100	99	Beihai, Guangxi	21.41175	109.162633	MG429105.1
13	GXBH-135	0.112	0.748	ThrMisF / ThrMisR	*Schizochytrium limacinum* (HM042913.2)	100	99	Beihai, Guangxi	21.41175	109.162633	MG429103.1
14	GXBH-215	0.110	0.751	Laby A / LabyY	*Labyrinthula* sp. (AB246794.1)	100	99	Beihai, Guangxi	21.41175	109.162633	MG429097.1
15	GXBH-233	0.102	0.713	ThrMisF / ThrMisR	*Schizochytrium limacinum* (HM042911.2)	99	99	Beihai, Guangxi	21.41175	109.162633	MG429115.1
16	GXBH-106	0.100	0.661	ThrMisF / ThrMisR	*Aurantiochytrium* sp. (KM023713.1)	86	100	Beihai, Guangxi	21.41175	109.162633	MG429099.1
17	GXBH-214	0.080	0.593	Laby A / LabyY	*Labyrinthula* sp. (AB246794.1)	100	100	Beihai, Guangxi	21.41175	109.162633	MG429096.1
18	GXBH-213	0.076	0.508	ThrMisF / ThrMisR	*Aurantiochytrium* sp. (KM023698.1)	100	99	Beihai, Guangxi	21.41175	109.162633	MG429109.1
19	GXBH-139	0.066	0.444	ThrMisF / ThrMisR	*Aurantiochytrium* sp. (JQ982490.1)	100	99	Beihai, Guangxi	21.41175	109.162633	MG429106.1
20	HNU-4	0.060	0.460	Laby A / LabyY	*Labyrinthula* sp. (AB246794.1)	100	99	Haikou, Hainan	20.06344	110.334516	MG429093.1
21	GXBH-229	0.057	0.261	Laby A / LabyY	*Aurantiochytrium* sp. (AB973504.1)	98	100	Beihai, Guangxi	21.41175	109.162633	MG429092.1
22	GXBH-219	0.055	0.414	Laby A / LabyY	*Labyrinthula* sp. (AB246794.1)	99	99	Beihai, Guangxi	21.41175	109.162633	MG429090.1
23	GXQZ-18	0.051	0.232	18S001 / 18S13	*Labyrinthula* sp. (FR875362.2)	100	100	Qinzhou, Guangxi	21.744233	108.59025	MG429079.1
24	GXQZ-21	0.050	0.252	ThrMisF / ThrMisR	*Aurantiochytrium* sp. (AB973502.1)	100	99	Qinzhou, Guangxi	21.744233	108.59025	MG429117.1

25	GXQZ-7	0.050	0.315	Laby A / LabyY	*Labyrinthula* sp. (FR875360.2)	100	100	Qinzhou, Guangxi	21.744233	108.59025	MG429098.1
26	FJLH-9	0.029	0.015	18S001 / 18S13	*Aurantiochytrium* sp. (KY977402.1)	100	99	Longhai, Fujian	24.438226	117.903722	MG429084.1
27	GXBH-108	0.012	0.889	ThrMisF / ThrMisR	*Schizochytrium* sp. (JQ248003.1)	100	99	Beihai, Guangxi	21.41175	109.162633	MG429100.1
28	GXBH-136	0.012	0.839	ThrMisF / ThrMisR	*Aurantiochytrium* sp. (JQ982492.1)	100	99	Beihai, Guangxi	21.41175	109.162633	MG429104.1
29	GXBH-209	0.012	0.810	ThrMisF / ThrMisR	*Aurantiochytrium* sp. (JQ982488.1)	86	99	Beihai, Guangxi	21.41175	109.162633	MG429108.1
30	GXBH-208	0.012	0.836	ThrMisF / ThrMisR	*Aurantiochytrium limacinum* (AB973564.1)	100	99	Beihai, Guangxi	21.41175	109.162633	MG429107.1
31	GXBH-114	0.011	0.826	ThrMisF / ThrMisR	*Thraustochytriidae* sp. (AY705757.1)	79	99	Beihai, Guangxi	21.41175	109.162633	MG429102.1
32	GXBH-223	0.011	0.801	18S001 / 18S13	*Aurantiochytrium* sp. (AB900161.1)	86	99	Beihai, Guangxi	21.41175	109.162633	MG429112.1
33	GXBH-224	0.011	0.794	ThrMisF / ThrMisR	*Aurantiochytrium* sp. (JX847358.1)	100	99	Beihai, Guangxi	21.41175	109.162633	MG429113.1
34	HNU-22	0.011	0.006	18S001 / 18S13	*Labyrinthula* sp. (FR875362.2)	100	100	Haikou, Hainan	20.06344	110.334516	MG429078.1
35	HNU-9	0.010	0.781	ThrMisF / ThrMisR	*Aurantiochytrium* sp. (KM023691.1)	100	100	Haikou, Hainan	20.06344	110.334516	MG429128.1
36	GXBH-205	0.010	0.687	Laby A / LabyY	*Labyrinthula* sp. (AB246794.1)	100	100	Beihai, Guangxi	21.41175	109.162633	MG429088.1
37	HNU-21	0.009	0.009	18S001 / 18S13	18S001 / 18S13	100	100	Haikou, Hainan	20.06344	110.334516	MG429077.1
38	HNHK-86	0.008	0.012	18S001 / 18S13	*Schizochytrium* sp.	100	99	Haikou,	20.062314	110.335232	MG429070.1

					(HQ228969.1)			Hainan			
39	HNU-5	0.008	0.011	18S001 / 18S13	*Labyrinthula* sp. (AB246795.1)	100	100	Haikou, Hainan	20.06344	110.334516	MG429076.1
40	HNHK-8	0.008	0.015	18S001 / 18S13	*Labyrinthula* sp. (FJ536742.1)	100	99	Haikou, Hainan	20.062314	110.335232	MG429065.1
41	HNHK-61	0.007	0.006	18S001 / 18S13	*Labyrinthula* sp. (FJ536742.1)	100	100	Haikou, Hainan	20.062314	110.335232	MG429067.1
42	HNU-18	0.007	0.014	Laby A / LabyY	*Labyrinthula* sp. (AB246794.1)	100	100	Haikou, Hainan	20.06344	110.334516	MG429094.1
43	HNHK-24	0.006	0.006	18S001 / 18S13	*Labyrinthula* sp. (AB246795.1)	100	100	Haikou, Hainan	20.062314	110.335232	MG429075.1
44	HNHK-75	0.005	0.004	18S001 / 18S13	*Schizochytrium* sp. (HQ228969.1)	100	99	Haikou, Hainan	20.062314	110.335232	MG429069.1
45	GXBH-101	0.004	0.006	Laby A / LabyY	*Labyrinthula* sp. (AB246794.1)	100	100	Beihai, Guangxi	21.41175	109.162633	MG429089.1
46	HNHK-12	0.003	0.004	18S001 / 18S13	*Parietichytrium* sp. (AB810977.1)	100	100	Haikou, Hainan	20.062314	110.335232	MG429073.1
47	HNHK-21	0.003	0.003	18S001 / 18S13	*Labyrinthula* sp. (AB246795.1)	100	100	Haikou, Hainan	20.062314	110.335232	MG429074.1
48	HNHK-4	0.000	0.000	18S001 / 18S13	*Labyrinthula* sp. (FJ536742.1)	100	100	Haikou, Hainan	20.062314	110.335232	MG429066.1
49	FJLH-1	ND	ND	18S001 / 18S13	*Aurantiochytrium* sp. (KY977402.1)	100	98	Longhai, Fujian	24.438226	117.903722	MG429085.1
50	GDSZ-1	ND	ND	18S001 / 18S13	*Thraustochytriidae* sp. (KU205263.1)	99	99	Shenzhen, Guangdo ng	22.520289	113.9496	MG429134.1
51	GDSZ-2	ND	ND	18S001 / 18S13	*Thraustochytriidae* sp. (AB973557.1)	100	100	Shenzhen, Guangdo	22.520289	113.9496	MG429135.1

								ng			
52	GXQZ-1	ND	ND	18S001 / 18S13	*Thraustochytrium aff. striatum* (HQ228973.1)	100	99	Qinzhou, Guangxi	21.744233	108.59025	MG429121.1
53	GXQZ-2	ND	ND	ThrMisF / ThrMisR	*Labyrinthula* sp. (AB290459.1)	100	97	Qinzhou, Guangxi	21.744233	108.59025	MG429127.1
54	GXQZ-10	ND	ND	ThrMisF / ThrMisR	*Aurantiochytrium* sp. (AB810944.1)	100	99	Qinzhou, Guangxi	21.744233	108.59025	MG429129.1
55	GXQZ-11	ND	ND	ThrMisF / ThrMisR	*Aurantiochytrium* sp. (AB973502.1)	97	99	Qinzhou, Guangxi	21.744233	108.59025	MG429122.1
56	GXQZ-12	ND	ND	18S001 / 18S13	*Labyrinthula* sp. (AB246795.1)	100	100	Qinzhou, Guangxi	21.744233	108.59025	MG429082.1
57	GXQZ-13	ND	ND	ThrMisF / ThrMisR	*Aurantiochytrium* sp. (AB973503.1)	100	100	Qinzhou, Guangxi	21.744233	108.59025	MG429130.1
58	GXQZ-17	ND	ND	18S001 / 18S13	*Labyrinthula* sp. (FR875362.2)	100	100	Qinzhou, Guangxi	21.744233	108.59025	MG429083.1
59	GXBH-102	ND	ND	ThrMisF / ThrMisR	*Schizochytrium limacinum* (HM042907.2)	100	99	Beihai, Guangxi	21.41175	109.162633	MG429123.1
60	GXBH-104	ND	ND	Laby A / LabyY	*Labyrinthula* sp. (AB246794.1)	100	100	Beihai, Guangxi	21.41175	109.162633	MG429086.1
61	GXBH-115	ND	ND	ThrMisF / ThrMisR	*Aurantiochytrium* sp. (JX847370.1)	100	99	Beihai, Guangxi	21.41175	109.162633	MG429126.1
62	GXBH-117	ND	ND	ThrMisF / ThrMisR	*Aurantiochytrium* sp. (KM023714.1)	100	99	Beihai, Guangxi	21.41175	109.162633	MG429125.1
63	GXBH-120	ND	ND	Laby A / LabyY	*Labyrinthula* sp. (AB246794.1)	100	100	Beihai, Guangxi	21.41175	109.162633	MG429095.1
64	HNHK-1	ND	ND	18S001 / 18S13	*Labyrinthula* sp.	100	100	Haikou,	20.062314	110.335232	MG429071.1

					(AB246794.1)			Hainan			
65	HNHK-10	ND	ND	18S001 / 18S13	*Labyrinthula* sp. (AB246795.1)	100	100	Haikou, Hainan	20.062314	110.335232	MG429072.1
66	HNHK-18	ND	ND	ThrMisF / ThrMisR	*Thraustochytriidae* sp. (AY705756.1)	100	100	Haikou, Hainan	20.062314	110.335232	MG429131.1
67	HNHK-36	ND	ND	18S001 / 18S13	*Labyrinthula* sp. (AB246795.1)	100	100	Haikou, Hainan	20.062314	110.335232	MG429080.1
68	HNHK-45	ND	ND	18S001 / 18S13	*Labyrinthula* sp. (AB246795.1)	100	100	Haikou, Hainan	20.062314	110.335232	MG429068.1
69	HNHK-68	ND	ND	18S001 / 18S13	*Labyrinthula* sp. (AB246795.1)	100	100	Haikou, Hainan	20.062314	110.335232	MG429081.1
70	HNHK-87	ND	ND	ThrMisF / ThrMisR	*Botryochytrium* sp. (AB973507.1)	100	100	Haikou, Hainan	20.062314	110.335232	MG429132.1
71	HNHK-88	ND	ND	18S001 / 18S13	*Parietichytrium* sp. (AB810977.1)	100	99	Haikou, Hainan	20.062314	110.335232	MG429133.1

ND: not determined.

Table S2. Primer-specific PCR programs employed for DNA amplification from isolated strains.

Primer pair	Primer sequence	PCR Program
18S001/18S13	18S001:<br>5′-AACCTGGTTGATCCTGCCAGTA-3′<br>18S13:<br>5′-CCITGTTACGACITCACCTTCCTCT-3′	Initial denaturation at 95 °C for 5 min, followed by 35 cycles of 1 min at 94 °C, 1 min at 50 °C and 1 min at 72 °C, and the final extension at 72 °C for 20 min.
ThrMisF/ThrMisR	ThrMisF:<br>5′-TGGGCAGCAGCACATGAG-3′<br>ThrMisR:<br>5′-GGTTCCTCGTTGGAGATT-3′	Initial denaturation at 95 °C for 5 min; 30 cycles of 30 s at 94 °C, 30 s at 52 °C and 60 s at 72 °C; and a final extension at 72 °C for 10 min
Laby-A/Laby-Y	Laby-A:<br>5′-GGGATCGAAGATGATTAG-3′<br>LABY-Y:<br>5′-CWCRAACTTCCTTCCGGT-3′	Initial denaturation at 95 °C for 3 min, followed by 33 cycles of 30 s at 95 °C, 30 s at 50 °C and 1 min at 72 °C, and the final extension at 72 °C for 20 min

Table S3. One-factor-at-a-time (OFAT) experimental design for optimization of DHA production in strain ZJWZ-7.

Design steps	Values of tested factors	Other factors
Step 1: Carbon source	*A. Single*<br>1. Glucose<br>2. Glycerol<br>3. Fructose<br>4. Mannose<br>Conc.: 20 g/L<br>*B. Combination*<br>1. Glucose (18g/L) - acetic acid (2g/L)<br>2. Glucose (18g/L) - ethanol (2g/L)<br>3. Glucose (18g/L) - citric acid (2g/L)<br>4. Glucose (18g/L) - malic acid (2g/L)<br>5. Glucose (18g/L) - potassium acetate (2g/L)<br>6. Glycerol (18g/L) - citric acid (2g/L)<br>7. Glycerol (18g/L) - malic acid (2g/L)<br>8. Glycerol (18g/L) - potassium acetate (2g/L)	Medium: 1.5 g/L peptone, 1 g/L YE, 0.25 g/L KH_2PO_4, 33 g/L artificial sea salt (100% artificial seawater), natural pH value;<br>Temperature: 28 °C;<br>Rotating rate: 150 rpm;<br>Inoculum size: 5 mL;<br>Seed age: 24 h;<br>Fermentation volume: 50 mL;<br>Fermentation time: 4 days.
Step 2: Nitrogen source	*A. Single*<br>1. Sodium glutamate<br>2. Tryptone<br>3. Peptone<br>4. Yeast extract<br>5. Ammonium sulfate<br>6. Ammonium nitrate<br>7. Sodium nitrate<br>8. Urea<br>Conc.: 2.5 g/L<br>*B. Combination*<br>9. Peptone (1.5 g/L) + yeast extract (1 g/L)	Medium: 20 g/L glucose, 0.25 g/L KH_2PO_4, 33 g/L artificial sea salt (100% artificial seawater), natural pH value;<br>Temperature: 28 °C;<br>Rotating rate: 150 rpm;<br>Inoculum size: 5 mL;<br>Seed age: 24 h;<br>Fermentation volume: 50 mL;<br>Fermentation time: 4 days.
Step 3: KH_2PO_4 concentration	0, 0.1, 0.25, 0.4 and 0.8 g/L	Medium: 20 g/L glucose, 1.5 g/L peptone, 1 g/L YE, 33 g/L artificial sea salt (50% artificial seawater), natural pH value;<br>Temperature: 28 °C;<br>Rotating rate: 150 rpm;<br>Inoculum size: 5 mL;<br>Seed age: 24 h;<br>Fermentation volume: 50 mL;<br>Fermentation time: 4 days.
Step 4: Salinity	0, 20, 40, 60, 80, 100 and 120 % SW	Medium: 20 g/L glucose, 1.5 g/L peptone, 1 g/L YE, 0.25 g/L

		KH_2PO_4, natural pH value;<br>Temperature: 28 °C;<br>Rotating rate: 150 rpm;<br>Inoculum size: 5 mL;<br>Seed age: 24 h;<br>Fermentation volume: 50 mL;<br>Fermentation time: 4 days.
Step 5: Temperature	20, 25, 28, 32 and 35°C	Medium: 20 g/L glucose, 1.5 g/L peptone, 1 g/L YE, 0.25 g/L KH_2PO_4, 33 g/L artificial sea salt (50% artificial seawater), natural pH value (pH 6.47);<br>Rotating rate: 150 rpm;<br>Inoculum size: 5 mL;<br>Seed age: 24 h;<br>Fermentation volume: 50 mL;<br>Fermentation time: 4 days.
Step 6: Initial pH	4, 5, 6, 6.47, 7 and 8 (adjusted by 4M H_2SO_4 and 4M NaOH)	Medium: 20 g/L glucose, 1.5 g/L peptone, 1 g/L YE, 0.25 g/L KH_2PO_4, 33 g/L artificial sea salt (50% artificial seawater);<br>Temperature: 28 °C;<br>Rotating rate: 150 rpm;<br>Inoculum size: 5 mL;<br>Seed age: 24 h;<br>Fermentation volume: 50 mL;<br>Fermentation time: 4 days.
Step 7: Rotating rate	100, 150, 180, 200 and 250 rpm	Medium: 20 g/L glucose, 1.5 g/L peptone, 1 g/L YE, 0.25 g/L KH_2PO_4, 33 g/L artificial sea salt (50% artificial seawater), natural pH value (pH 6.47);<br>Temperature: 28 °C;<br>Inoculum size: 5 mL;<br>Seed age: 24 h;<br>Fermentation volume: 50 mL;<br>Fermentation time: 4 days.
